# Igneous phosphate rock solubilization by biofilm-forming mycorrhizobacteria and hyphobacteria associated with *Rhizoglomus irregulare* DAOM 197198

**DOI:** 10.1007/s00572-016-0726-z

**Published:** 2016-08-19

**Authors:** Salma Taktek, Marc St-Arnaud, Yves Piché, J. André Fortin, Hani Antoun

**Affiliations:** 1 Centre de recherche en innovation sur les végétaux and Département des sols et de génie agroalimentaire, Faculté des sciences de l’agriculture et de l’alimentation, Université Laval, 2480 Boulevard Hochelaga, Québec, QC G1V 0A6 Canada; 2 Institut de recherche en biologie végétale, Université de Montréal and Jardin botanique de Montréal, Montréal, H1X 2B2 QC Canada; 3 Centre d’étude de la forêt and Département des sciences du bois et de la forêt, Faculté de foresterie, de géographie et de géomatique, Université Laval, Québec, G1V 0A6 QC Canada

**Keywords:** AMF-PSB interactions, *Burkholderia* biofilm, Phosphate rock solubilization, *Rhizoglomus irregulare* hyphae

## Abstract

Biofilm formation on abiotic and biotic surfaces was studied with two hyphobacteria, strongly attached to the surface of the arbuscular mycorrhizal fungus (AMF) *Rhizoglomus irregulare* (*Ri*) DAOM 197198 and two mycorrhizobacteria, loosely attached to the roots of different mycorrhizal plants. When the sparingly soluble igneous phosphate rock (PR) from Quebec, or when the chemical hydroxyapatite were used as sole phosphorus (P) source, hyphobacteria *Rhizobium miluonense* Rm3 and *Burkholderia anthina* Ba8 produced significantly more biofilms than mycorrhizobacteria *Rahnella* sp. Rs11 and *Burkholderia* *phenazinium* Bph12, as indicated by the crystal violet assay or by quantifying biofilm exopolysaccharides. As previously observed with planktonic bacteria, biofilms mobilized P by lowering the pH and releasing gluconic acid. The high efficiency of P mobilization by the hyphobacteria Ba8 was linked to the presence of more viable cells in its biofilm as revealed by the hydrolysis of fluorescein diacetate. Scanning electron microscopy micrographs showed a high adherence of the best P-solubilizer hyphobacteria Ba8 on the surface of Quebec PR. Hydroxyapatite porous structure did not allow a good adherence of Ba8. Ba8 formed an important biofilm on the hyphae of *Ri* DAOM 197198 with low reactive Quebec PR while no biofilm was observed with the high reactive hydroxyapatite. Results confirm the possible presence of specificity between the *Ri* DAOM 197198 and the hyphobacteria and suggest that the interaction would be regulated by the availability of P.

**Electronic supplementary material:**

The online version of this article (doi:10.1007/s00572-016-0726-z) contains supplementary material, which is available to authorized users.

## Introduction

Although total phosphorus (P) may be abundant in soils in inorganic and organic forms, it is an important plant growth-limiting nutrient (Gyaneshwar et al. 2002). In fact, plants require large amount of soluble orthophosphate for growth, but the concentration of these P anions in soil solution is very low, because they rapidly react with available cations to form sparingly soluble calcium phosphates in alkaline soils or iron and aluminum phosphates in acid soils (Gyaneshwar et al. 2002; Richardson 2001; Whitelaw 2000). Water-soluble P fertilizers, like triple superphosphate or ammonium phosphates, are usually used to compensate for soil deficiency in soluble P, but their use on a regular basis is costly and may produce potential environmental problems like eutrophication caused by the slow flux of P from over-fertilized soils (Carpenter 2008). Therefore, it is necessary to develop economically and ecofriendly technologies that will allow the mobilization of P from soil reserves. Phosphate rock (PR) deposits are a cheap source of phosphate fertilizer (Reddy et al. 2002) and compared to the soluble dipotassium phosphate, the application of a PR-based fertilizer to an acidic soil significantly reduced losses of P and other trace elements in leachates (Yang et al. 2013). Therefore the direct use of PR appears to be environmentally friendly; however, application of untreated PR as fertilizer did not show great successes, mainly because most available PR worldwide have low reactivity (Vassilev et al. 2001).

Soil microorganisms play a key role in soil functional processes including organic matter decomposition, turnover, and release of P (Van der Heijden et al. 2008). In natural terrestrial plant ecosystems, phosphate-solubilizing microorganisms (PSM) and mycorrhizal fungi are responsible for most P acquisition by plants. Dissolution (mobilization) of inorganic P by PSM is performed by the release of low molecular weight organic acids or protons, while microbial enzymes are responsible for the mineralization and hydrolysis of organic P (Richardson and Simpson 2011). Phosphate-solubilizing bacteria (PSB) are usually present in the rhizosphere rich in the nutrients released by root exudates (Rodriguez and Fraga 1999). However, the response of cultivated plants to inoculation with PSB is erratic, and there is very little evidence in the literature clearly showing that inoculation with PSB alone increases plants P uptake (Pii et al. 2015). This can be attributed not only to the nature of the soil used but also to the diversity of fauna and microorganisms including mycorrhizal fungi that interact with the introduced PSB (Antoun 2012). In fact, many reports clearly illustrate the enhancement of P mobilization by arbuscular mycorrhizal fungi (AMF) in the presence of PSB, in addition to other synergistic interactions between AMF and rhizobacteria (for a review see Artursson et al. (2006)). Furthermore, based on the literature, Bonfante and Anca (2009) suggested that bacteria loosely or strongly attached to arbuscular mycorrhiza (AM) hyphae are probably third partners playing a major role in the plant-mycorrhiza symbioses. Considering the large bacterial diversity found in the mycorrhizosphere and attached to AM hyphae, they also suggested the presence of bacterium/mycorrhizal fungus specificity.

We have recently described a method to isolate phosphate-solubilizing hyphobacteria strongly attached to the hyphae of the AM fungus *Rhizoglomus irregulare* DAOM 197198 (*Ri*; formerly *Glomus irregulare* or *Rhizophagus irregularis*; Sieverding et al. 2014) and observed that in a two-compartment Petri plate system, the extraradical *Ri* hyphal exudates supported the growth of PSB (Taktek et al. 2015). Under these experimental conditions, *Ri* hyphae mobilized very little P from PR; however, in the presence of PSB, substantially more P was mobilized. In addition, we found that hyphobacteria *Burkholderia anthina* Ba8 and *Rhizobium miluonense* Rm3, in general, showed higher P mobilization activity than mycorrhizobacteria *Rahnella* sp. RS11 and *Burkholderia phenazinium* Bph12 isolated from the mycorrhizosphere (loosely attached to the rhizosphere and AMF hyphae of mycorrhizal plants).

AMF hyphal network provides an important source of recent plant photosynthates used by different soil microbes (Kaiser et al. 2015) and biological surfaces for interactions between AMF and soil bacteria, especially PSB (Frey-Klett et al. 2007; Johansson et al. 2004). In the mycorrhizosphere, certain bacteria are able to attach to abiotic (soil particles) and biotic surfaces (roots and AM hyphae) and form biofilms (Seneviratne et al. 2008), some of which have plant growth-promoting traits (for review see Ramey et al. 2004). The bacterial communities forming biofilms are characterized by an irreversible attachment to certain surfaces involving a distinct set of molecular mechanisms and stimuli (Donlan and Costerton 2002). Microbial biofilms are involved in the biogeochemical cycles of many elements, and bacteria cooperate within a biofilm matrix rather than in a planktonic state (Davey and O’Toole 2000). In fact, many genes that are not transcribed by planktonic organisms will be expressed when the organisms are forming biofilms (Donlan and Costerton 2002). Fungal-bacterial biofilms exhibited higher metabolic activities compared to monocultures (Seneviratne et al. 2008). Colonization of the hyphae of the soil fungus *Penicillum* sp. by *Bradyrhizobium elkani* SEMIA 5019 produced a biofilm that significantly enhanced P mobilization from a Sri Lankan PR (Jayasinghearachchi and Seneviratne 2006).

Many associations between AMF and soil bacteria have important implications in agriculture by enhancing nutrients uptake, namely P (Miransari 2011). Since hyphobacteria strongly attached to *Ri* DAOM 197198 hyphae solubilized more efficiently a low reactive igneous PR from Québec Canada than loosely attached mycorrhizobacteria (Taktek et al. 2015), we hypothesized that the superiority of hyphobacteria can be attributed to their ability to form important biofilms with high P mobilizing activity. Here, we describe bacterial biofilms formed on abiotic surfaces by hyphobacteria trapped on *Ri* hyphae and by mycorrhizobacteria isolated from roots of different mycorrhizal plants, and we compare their P mobilizing activities from a Quebec PR or hydroxyapatite. Biofilm formed by hyphobacteriun *B. anthina* Ba8 on hyphae of *Ri* DAOM197198 is also described.

## Materials and methods

### Bacterial strains and mycorrhizal fungus

Four PSB were used in this work: hyphobacteria *R. miluonense* Rm3 (GenBank KC241902) and *B. anthina* Ba8 (GenBank KC241903) trapped on hyphae of the AMF *Ri* DAOM 197198, and mycorrhizobacteria *Rahnella* sp. Rs11 (GenBank KC241900) and *B. phenazinium* Bph12 (GenBank KC241901) isolated from the mycorrhizosphere of different mycorrhizal plants (Taktek et al. 2015). Spores of *Ri* DAOM 197198 were kindly supplied by Premier Tech, Rivière-du-Loup, Québec, Canada.

### Evaluation of bacterial biofilms formation on sparingly soluble phosphate sources

Biofilm formation by PSB was appraised by three different methods in microtiter plates. In a preliminary study, we determined the ability of PSB to mobilize P under biofilm formation at different time intervals (1, 3, 5, and 7 days). Results indicated that the optimum biofilm formation was obtained in flat bottom 96- or 6-well polystyrene microtiter plates after 5 days incubation at 28 °C without agitation. This period of time also allowed maximum P mobilization from Quebec PR ground to 150 μm (Taktek et al. 2015) or hydroxyapatite. All tests were performed in a completely randomized block design with four to nine blocks as specified below by using as sole P source either the low reactive Quebec igneous PR (total P 146.44 mg g^−1^; 4.97 mg g^−1^ soluble in 2 % formic acid; and 7.05 mg g^−1^ soluble in 2 % citric acid) or the hydroxyapatite, a synthetic micropowder of calcium apatite (Sigma-Aldrich; 20–50 μm) used as a control for its high reactivity.

#### Crystal violet assay

A crystal violet (CV) assay was performed to estimate biofilm formation as previously described (O’Toole 2011) with slight modifications. Inocula were prepared by growing bacteria overnight on a rotary shaker (180 rpm) at room temperature in 25 mL of 10 % tryptic soy broth (TSB; Becton, Dickinson and Co., Franklin Lakes, NJ, USA). Cells were collected and washed twice in 8 mL sterile normal saline (SNS; 0.85 % NaCl) by centrifugation (10,000×*g* for 5 min at 4 °C) and resuspended in 8 mL SNS to give more than 10^8^ CFU mL^−1^. Flat bottom wells of sterile polystyrene 96-well microtiter plates (Costar®, Corning Inc. Tewksbury, MA, USA) were filled with 100 μL of the modified National Botanical Research Institute’s Phosphate medium (NBRIP; Nautiyal 1999) containing per liter of sterile water: 5 g MgCl_2_·6H_2_O, 0.25 g MgSO_4_·H_2_O, 0.2 g KCl, 0.1 g (NH_4_)_2_SO_4_, 10 g glucose, and 5 g of Quebec PR or hydroxyapatite. Each well was inoculated with 10 μL of inoculum containing 10^6^ CFU. Control wells received 10 μL SNS without bacteria. After 5 days of biofilm formation at 28 °C, the supernatant was removed and the wells were rinsed twice with 100 μL of SNS, air dried for 30 min, and then 100 μL of a 0.1 % solution of CV in water was added. After 15 min of incubation at room temperature, the excess of CV was removed by gently washing the plates 3–4 times with running tap water and then microtiter plates were turned upside down and dried. Finally, bound CV was solubilized by adding 100 μL of 30 % acetic acid in water and microtiter plates were incubated for 15 min. The absorbance was measured at 550 nm on a Biochrom Asys UVM 340 microplate reader (Biochrom Ltd., Cambridge, UK). All treatments were replicated nine times.

In this assay, the extent of biofilm formation was determined by applying the following formula: BF = AB − CW, where BF is the biofilm formation, AB is the optical density (OD_550nm_) of stained attached bacteria, and CW is the OD_550nm_ of stained background color containing bacterial-free medium only (Kadurugamuwa et al. 2003; Naves et al. 2008).

#### Exopolysaccharides assay

For biofilm estimation by the exopolysaccharides (EPS) assay, wells of flat bottom sterile polystyrene six-well microtiter plate (Costar®, Sigma-Aldrich) containing 8 mL of modified NBRIP medium were inoculated with 1 mL of the inoculum containing 10^7^ CFU mL^−1^, prepared as described earlier. Control wells received 1 mL of SNS without bacteria. The six-well plates were incubated at 28 °C without agitation during 5 days. Biofilm determination was done by quantifying the amount of EPS in the biofilm matrix by using the method of Dubois et al. (1956) as modified by Furtner et al. (2007). Briefly, the supernatant was removed, and the wells were rinsed twice with SNS, then 4.42 mL of a mixture of 0.23 % (*v*/*v*) formaldehyde (Sigma-Aldrich) in SNS was added. The plates were agitated 5 min at 80 rpm on a rotatory shaker at room temperature and then incubated for 1 h at 4 °C. After incubation, 1.76 mL of a 1 M solution of NaOH was added, and the mixtures were incubated at room temperature for 10 min. Eppendorf® tubes containing 1.5 mL of each sample were centrifuged at 10,000×*g* at 4 °C for 1 min, then 800 μL of the supernatant was recovered in a glass tube and mixed with 800 μL of a 5 % phenol solution (*v*/*v*), and then 4 mL of 96.9 % (*v*/*v*) H_2_SO_4_ was rapidly added. The mixture was cooled in ice for 20 min, and OD_448nm_ was determined. All treatments were replicated four times. The amount of EPS was determined using a standard curve (0–0.2 mg L^−1^) of d-glucose (Sigma-Aldrich).

#### Fluorescein diacetate assay

Fluorescein diacetate (FDA) assay was used to quantify physiologically active cells forming the biofilm, based on the ability of microbial enzymes (e.g., esterases, lipases, proteases) to split the FDA molecule, producing fluorescein. Culture conditions have been set as previously described in CV assay section. After 5 days of adhesion, the supernatant was removed and the wells were rinsed with 100 μL of 0.1 M 3-(*N*-morpholino) propanesulfonic acid (MOPS) buffer. One liter MOPS buffer contains 20.9 g MOPS and 5.6 g NaCl, pH 7.00.

FDA was dissolved in acetone at a concentration of 10 mg mL^−1^, and this stock solution was stored at −20 °C. A 1:50 FDA working solution was freshly prepared in MOPS buffer before each assay. MOPS buffer (100 μL) was added to each rinsed well, followed by the addition of 100 μL FDA working solution. Plates were incubated in the dark at 37 °C, and fluorescence was measured after 30 min at 490 nm. All treatments were replicated four times. A standard curve was prepared using fluorescein sodium salt in acetone (Peeters et al. 2008).

#### Phosphate solubilization and organic acids production under biofilm formation

Cultures were prepared as described previously in six-well microtiter plates (Costar®) (see the “Exopolysaccharides assay” section). After 5 days of incubation at 28 °C without agitation on modified NBRIP, aliquots were removed from each well to determine the amount of soluble P released and the pH. The amount of soluble P was determined as described by Taktek et al. (2015). Briefly, 100 μL of the supernatant was diluted in an appropriate volume of distilled water and mixed with 100 μL of 5.5 % (*w*/*v*) trichloroacetic acid (Sigma-Aldrich) and incubated for 10 min. Then, 100 μL of molybdate solution (prepared by mixing 40 mL of 1.5 % (w/v) ammonium molybdate in 5.5 % (v/v) H_2_SO_4_, with 10 mL of 2.7 % (w/v) FeSO_4_.6H_2_O in distilled water) were added and the reaction mix was incubated for 15 min in the dark. The absorbance was measured at 700 nm in a Biochrome Asys UVM340 microplate reader. Phosphate concentration was determined using a KH_2_PO_4_ standard curve. All treatments were replicated 12 times. To measure organic acids, the remaining supernatant from the bacterial cultures of three randomly chosen replicates was filtered through 0.2 μm filter (Millipore) and 10 μL of filtrates was separated on a Waters HPLC system (Pump Model 996) using a ICSep ICE-ION-300 column (300 × 7.8 mm) with an isocratic solvent (8.5 mM H_2_SO_4_) at a flow of 0.4 mL min^−1^.

### Scanning electron microscopy

#### Bacterial attachment to abiotic surface

Biofilms of *B. anthina* Ba8 developed on the surface of phosphate particles, as described above, were fixed with 2.5 % (*v*/*v*) glutaraldehyde in 0.1 M sodium cacodylate buffer at pH 7.3 for 24 h at room temperature. They were then washed three times with sodium cacodylate buffer for 30 min, treated with 1 % osmium tetroxide in sodium cacodylate buffer for 90 min, and washed again with sodium cacodylate buffer. The samples were subsequently dehydrated for 10 min in each concentration of a series of ethanol solutions (50, 70, 95, and 100 %) and treated twice in 100 % ethanol for 40 and 10 min and then twice with hexamethyldisilazane for 30 min. Samples were then air dried, coated with gold, and viewed under a JEOL 6360LV scanning electron microscope (SEM) (Tokyo, Japan) in high vacuum mode at 25 kV. All treatments were replicated three times.

#### Bacterial attachment to biotic surface

*B. anthina* Ba8 bacterial cells attachment to AMF hyphae was evaluated in two-compartment plates, which consist of a proximal root-AMF compartment and a distal hyphal only compartment (St-Arnaud et al. 1995). Initially, AMF *Ri* DAOM 197198 was grown on chicory (*Cichorium intybus*) roots transformed with the tumor-inducing plasmid T-DNA at 28 °C in a minimal growth medium (Taktek et al. 2015). When extensive amount of extraradical AMF *Ri* hyphae colonized the distal compartment (after approximately 3 weeks), a 1-cm wide hyphal zone was kept in the distal compartment (Fig. S1, supplementary material) and the rest was removed and replaced with 20 mL of a liquid minimum medium containing (mg L^−1^): 80 KNO_3_, 731 MgSO_4_·7H_2_O, 65 KCl, 4.8 KH_2_PO_4_, 288 Ca(NO_3_)_2_·4H_2_O, 8 NaFeEDTA, 0.75 KI, 6 MnCl_2_·4H_2_O, 2.65 ZnSO_4_·7H_2_O, 1.5 H_2_BO_3_, 0.13 CuSO_4_·5H_2_O, and 0.0024 Na_2_MoO_4_·2H_2_O. The bacterial adherence to AMF *Ri* hyphae was tested with KH_2_PO_4_ as solid phase-free control and by replacing KH_2_PO_4_ in the minimum medium with 2500 mg L^−1^ of two solid phosphate sources of low or high reactivity, respectively Quebec PR (ground to <150 μm) and hydroxyapatite. When the AMF *Ri* hyphae had colonized the liquid compartment (after approximately 3 weeks,), 10 μL of bacterial suspension containing 10^6^ CFU was added and plates were incubated at 28 °C for 6 weeks. Hyphae with attached bacteria were collected, fixed (as described in the previous section), and observed under a JEOL 6360LV SEM (Tokyo, Japan) in high vacuum mode at 25 kV. All treatments were replicated three times.

### Statistical analysis

Normality of the residuals and homogeneity of variance were verified for all treatments using the MIXED procedure (PROC MIXED) of the SAS 9.2 software. Box-Cox transformations were used to ensure the normality assumption in a linear regression model for CV and FDA assays, P-solubilization quantification, and also for organic acids produced on NBRIP medium containing hydroxyapatite. Means were compared using the Fisher’s protected least significant difference method at *P* ≥ 0.05.

## Results

### Biofilm quantification

The bacterial biofilm development differed significantly (*P* ≤ 0.05) among PSB strains (Table 1) and its appraisal was influenced by the quantification method used. The CV method suggests that PSB formed comparable biofilms in the presence of Quebec PR or hydroxyapatite. However, according to the EPS method, which quantifies a major component of the biofilm matrix, biofilms formed in the presence of Quebec PR were about four times larger than those formed in the presence of hydroxyapatite (Table 1). In addition, EPS measurements indicated that the two hyphobacteria *R. miluonense* Rm3 and *B. anthina* Ba8 formed significantly larger biofilms than the two mycorrhizobacteria *Rahnella* sp. Rs11 and *B. phenazinium* Bph12 in the presence of both sources of P (Table 1). Both CV and EPS methods revealed that hyphobacterium Rm3 was the major biofilm producer, and in general, the importance of biofilm formation by PSB tends to follow the pattern: Rm3 > Ba8 > Bph12 > Rs11. The FDA assay showed that the hyphobacterium Ba8 formed biofilms containing the highest amount of viable and active cells in the presence of Quebec PR or hydroxyapatite.

**Table 1:** P-solubilization, pH of supernatants, and biofilm formation by phosphate solubilizing bacteria after 5 days of incubation in liquid modified NBRIP containing Quebec phosphate rock (PR) or hydroxyapatite as sole source of P

Phosphate solubilizing bacteria	P-solubilization and pH of the supernatants^a^		Biofilm assay^a^		
	P (mg L^−1^)	pH	Crystal violet (BF^b^)	EPS (μg mL^−1^)	FDA (μg mL^−1^ h^−1^)
	Quebec PR				
Hyphobacteria					
Rm3	4.18 ± 0.21c	3.01 ± 0.02b	0.65 ± 0.21a	40.15 ± 1.13a	0.44 ± 0.03c
Ba8	8.27 ± 0.51a	2.82 ± 0.01d	0.44 ± 0.13bc	35.96 ± 1.30b	1.99 ± 0.11a
Mycorrhizobacteria					
Rs11	3.69 ± 0.81d	3.34 ± 0.04a	0.35 ± 0.07c	29.20 ± 0.79d	0.35 ± 0.03d
Bph12	5.75 ± 0.71b	2.92 ± 0.05c	0.46 ± 0.10b	33.35 ± 1.62c	0.63 ± 0.03b
	Hydroxyapatite				
Hyphobacteria					
Rm3	94.14 ± 2.96b	3.81 ± 0.05b	0.43 ± 0.04a	11.03 ± 0.73a	0.51 ± 0.05b
Ba8	129.35 ± 8.6a	3.30 ± 0.02d	0.35 ± 0.03b	7.39 ± 0.22b	0.67 ± 0.07a
Mycorrhizobacteria					
Rs11	43.72 ± 2.57d	3.63 ± 0.02c	0.31 ± 0.03c	4.31 ± 0.24c	0.31 ± 0.05d
Bph12	65.98 ± 3.19c	4.10 ± 0.07a	0.32 ± 0.03bc	7.27 ± 0.08b	0.41 ± 0.03c

For each P source, means in each column followed by the same letter are not significantly different according to Fisher’s protected LSD test (*P* ≤ 0.05). No soluble P was detected in non-inoculated control treatments and their culture medium had a pH = 7

*Rm3 Rhizobium miluonense*, *Ba8 Burkholderia anthina*, *Rs11 Rahnella* sp., *Bph12 Burkholderia phenazinium*

^a^Values are means ± standard deviations for P and pH (*n* = 12), crystal violet (*n* = 9), exopolysaccharides (EPS; *n* = 4), and fluorescein diacetate (FDA; *n* = 4)

^b^BF = AB − CW. *BF* biofilm formation, *AB* stained attached bacteria, *CW* stained control wells

### P-solubilization and organic acids production under biofilm formation

Under cultural conditions promoting the formation of biofilms, hyphobacteria in general, mobilized significantly more P from PR or hydroxyapatite than mycorrhizobacteria (Table 1). In fact, after 5 days of incubation without agitation in microtiter plates, the highest P-solubilization activity was observed with hyphobacterium *B. anthina* Ba8 biofilm formed in NBRIP medium supplemented with PR (8.3 mg P L^−1^) or hydroxyapatite (129.4 mg P L^−1^) as sole source of P. In the presence of both sources of P, mycorrhizobacterium *Rahnella* sp. Rs11 exhibited the lowest P mobilization activity (Table 1).

P-solubilization was accompanied by a significant drop in the pH of supernatants (Table 1) as compared to the uninoculated treatment (pH 7). The lowest pH values were observed when the bacteria were grown with Quebec PR, with the lowest pH value (2.8) obtained with the best P-solubilizer *B. anthina* Ba8. When hydroxyapatite was used as sole P source, the pH values ranged between 3.3 and 4.1 and as observed with PR, hyphobacterium *B. anthina* Ba8 produced more acidity (pH 3.3) than other PSB (Table 1). In the uninoculated control treatments, no soluble-P and no decrease in the pH were detected.

Results showed that mobilization of P from sparingly soluble sources by PSB biofilms was accompanied by the production of gluconic and 2-ketogluconic acids (Table 2). Using NBRIP-Quebec PR medium, *B. anthina* Ba8 was the best producer of gluconic acid (1172.4 mg L^−1^) followed by Bph12 and Rm3, while this organic acid was not detected with Rs11. The 2-ketogluconic acid was only produced by hyphobacteria Rm3 and Ba8 in similar amounts (Table 2). In NBRIP-hydroxyapatite medium, *R. miluonense* Rm3, *B. anthina* Ba8, and *Rahnella* sp. Rs11 produced both gluconic acid and 2-ketogluconic acid, whereas Bph12 produced only gluconic acid (Table 2). The amounts of organic acids differed among bacterial strains, Rs11 producing the highest amount of gluconic acid, while Ba8 was the higher producer of ketogluconic acid. While the link between the organic acid release and P-solubilization was more clear when Quebec PR was used as P source, the best organic acid producer (Rs11) was the poorest P-solubilizer with hydroxyapatite.

**Table 2:** Organic acid production by biofilm-forming phosphate solubilizing bacteria after 5 days of incubation in liquid modified NBRIP containing Quebec phosphate rock (PR) or hydroxyapatite as sole source of P

Phosphate solubilizing bacteria	Organic acid (mg L^−1^)^a^	
	Gluconic acid	2-Ketogluconic acid
	Quebec PR	
Hyphobacteria		
Rm3	277.8 ± 12.3c	237.7 ± 5.1a
Ba8	1172.4 ± 26.3a	226.08 ± 8a
Mycorrhizobacteria		
Rs11	–	–
Bph12	774.5 ± 38.8b	–
	Hydroxyapatite	
Hyphobacteria		
Rm3	364.1 ± 69.1b	200 ± 2.7b
Ba8	110.3 ± 8.2c	1226.7 ± 22.6a
Mycorrhizobacteria		
Rs11	2041.9 ± 200.6a	210.4 ± 10.1b
Bph12	409.4 ± 21.9b	–

*Rm3 Rhizobium miluonense*, *Ba8 Burkholderia anthina*, *Rs11 Rahnella* sp., *Bph12 Burkholderia phenazinium*, *–* not detected

^a^Values are means ± standard deviations (*n* = 3). For each P source, means in each column followed by the same letter are not significantly different according to Fisher’s protected LSD test (*P* ≤ 0.05)

### Biofilm scanning electron microscopy analysis

**Fig. 1:**
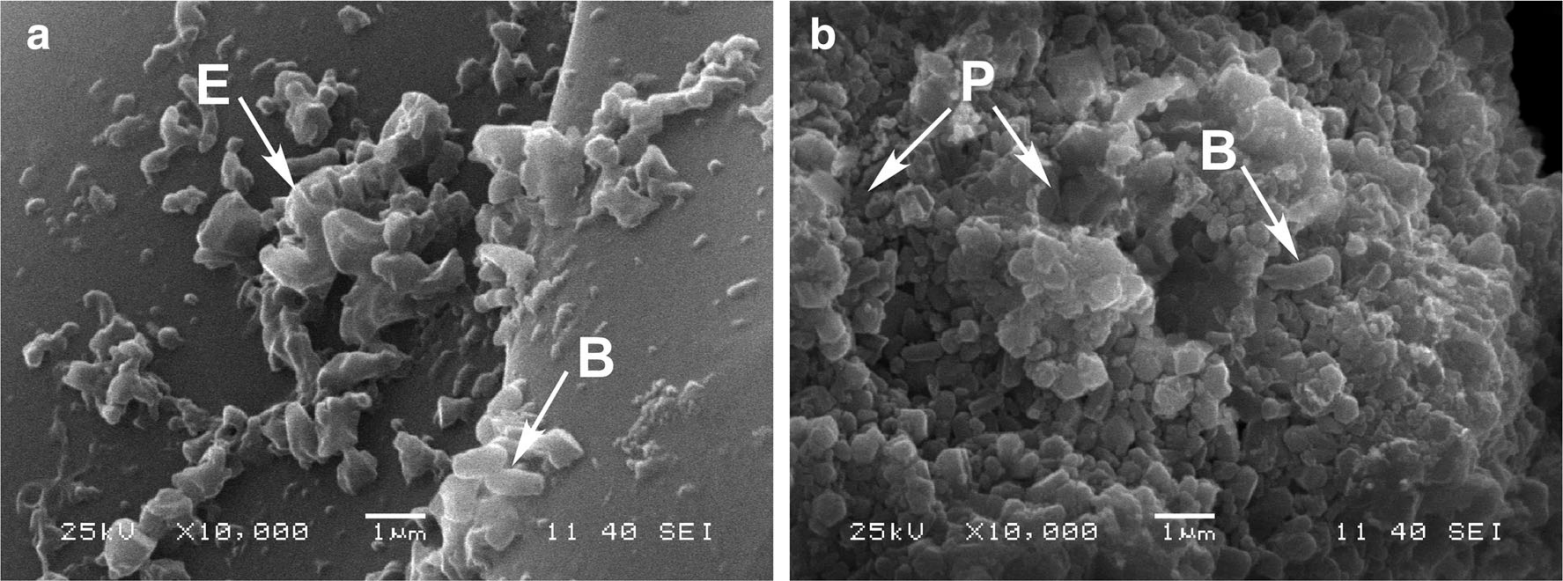
Scanning electron micrographs of *Burkholderia anthina* Ba8 bacterial matrix formed on sparingly soluble phosphates after 5 days of incubation. Micrographs show exopolysaccharides (*E*) connecting bacterial cell (*B*) to a particle of **a** Quebec phosphate rock or **b** bacterial cells (*B*) on the pores (*P*) of hydroxyapatite

Since the hyphobacterium *B. anthina* Ba8 formed the best viable biofilm on phosphate particles as revealed by the FDA assay, it was further examined using SEM. Representative SEM images of Ba8 growing in NBRIP media with either Quebec PR or hydroxyapatite as sole P source are presented in Fig. 1. Ba8 showed an extensive growth on Quebec PR surface as compared to hydroxyapatite surface. Cell aggregates were held together and protected by an extracellualar matrix of secreted polymeric compound (EPS). A three-dimensional structure formed of bacterial cells and EPS was observed over Quebec PR particles (Fig. 1a), while it was less developed on hydroxyapatite surfaces (Fig. 1b). SEM micrographs also showed that the surface of Quebec PR was regular and smooth while that of hydroxyapatite was rough and porous.

**Fig. 2:**
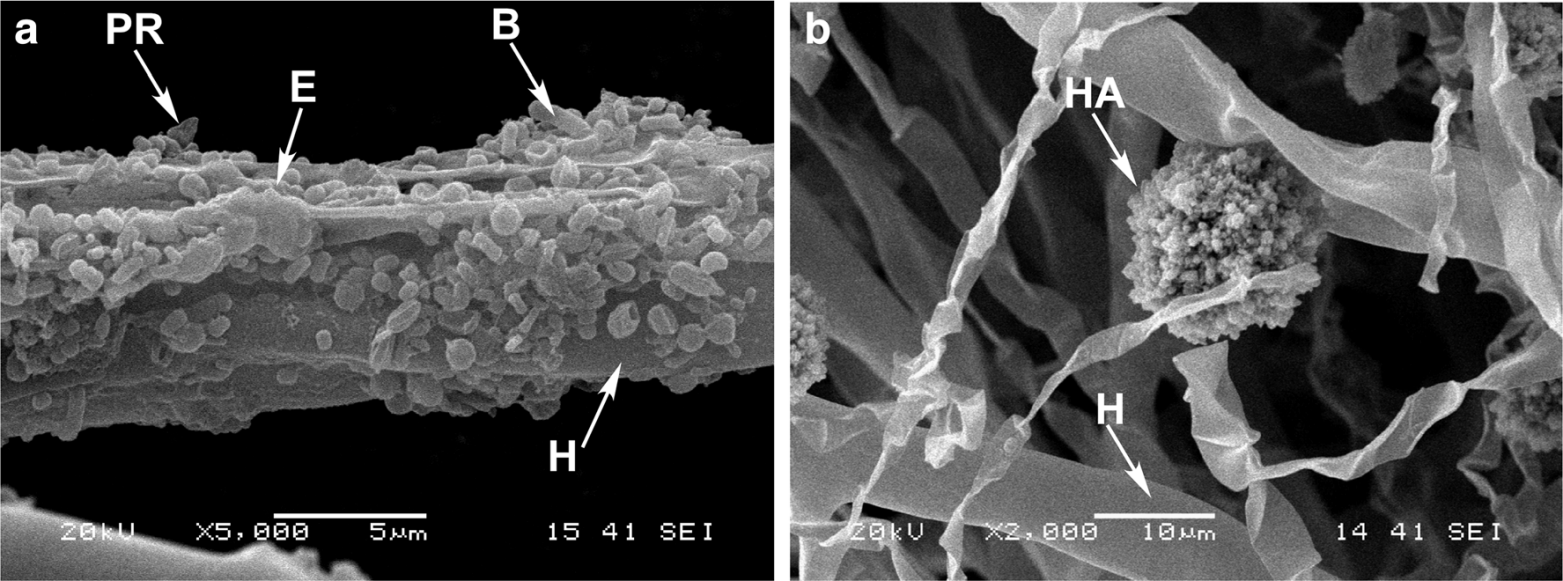
Scanning electron micrographs of *Burkholderia anthina* (Ba8) bacterial matrix formed on *Rhizoglomus irregulare* DAOM197198 hyphae using **a** Quebec phosphate rock (PR) or **b** hydroxyapatite (HA) after 6 weeks of dual culture. Micrographs show **a** exopolysaccharides (*E*) and bacterial cells (*B*) constituting the biofilm formed on hyphae (*H*); **b** absence of bacterial cells attachment on hyphae (*H*) in the vicinity of hydroxyapatite (*HA*)

Hyphobacterium *B. anthina* Ba8 was not only the best biofilm-forming bacterium on phosphate particles but it also presented an obvious attachment to AMF *Ri* extraradical mycelium that was clearly visible when Quebec PR was used as P source (Fig. 2a). Micrographs showed that EPS connected bacterial cells and small particles of Quebec PR on the surface of AMF *Ri* hyphae. However, no bacterial adherence was observed when the high reactivity hydroxyapatite (Fig. 2b) or the soluble KH_2_PO_4_ was used as sole P source (results not shown).

## Discussion

In plant-microbe associations, the biofilms formed can have detrimental or beneficial effects on plant growth and health (Ramey et al. 2004). Development of beneficial biofilms able to promote plant nutrition, growth and health can therefore play a significant role in the development of sustainable agricultural practices by reducing the use of fertilizers and pesticides. There is a general agreement that biofilm formation is merely defined as sticky bacterial cells attached to surfaces whatever their structure or microbial compositions (Berne et al. 2010). We have previously demonstrated that phosphate-solubilizing hyphobacteria strongly attached to the hyphae of AMF *Ri* associated with leek plants were more likely successful to mobilize P from a Quebec PR than the loosely attached mycorrhizobacteria (Taktek et al. 2015). We hypothesized that strongly attached hyphobacteria probably form more important biofilms on abiotic and biotic surfaces compared to the loosely attached mycorrhizobacteria. Here, we investigated the formation of biofilms formed by hyphobacteria and mycorrhizobacteria and showed that the amount of viable cells in the biofilm formed is correlated with their phosphate mobilization activity.

Different assays have been used to estimate the abundance of biofilms formation in microtiter plates, namely the CV staining, the EPS matrix quantification, and the FDA viability assay. CV staining and EPS assay showed that the hyphobacteria tend to form more important biofilms than the mycorrhizobacteria. This can explain in part why the isolated hyphobacteria adhere to *Ri* hyphae more strongly than mycorrhizobacteria. As revealed by CV and EPS assays, in the presence of both P sources, biofilms formed by hyphobacterium *R. miluonense* (Rm3) were significantly more important than those formed by other PSB. In fact, rhizobia are known to be good biofilm formers both on abiotic surfaces and roots (Rinaudi et al. 2006) and on the hyphae of common soil fungi (Jayasinghearachchi and Seneviratne 2006).

Since CV and EPS tests also measure dead and inactive biomass (Peeters et al. 2008), to appraise the P mobilization potential of PSB biofilms, it is important to determine their content of viable and metabolically active cells. This was performed by using the hydrolysis of fluorescein diacetate (FDA) test designed to account for metabolically active cells only (Borucki et al. 2003; Peeters et al. 2008). FDA measurements showed that biofilms formed by hyphobacteria Ba8 and Rm3 contained significantly more viable cells than biofilms formed by mycorrhizobacteria, with a maximum viable biofilm formed by the phosphate-solubilizing hyphobacterium *B. anthina* Ba8 (Table 1). All PSB biofilms were able to solubilize inorganic phosphates to reduce pH and to release organic acids. Higher levels of solubilized P were obtained with *B. anthina* Ba8 compared to the other strains. In fact, this strain caused the highest drop in pH and had the highest content in viable cells. Accordingly, P-solubilizing ability of biofilms appeared to be correlated with cells viability and not to biofilm strength. Then, the lower amounts of solubilized P obtained with *R. miluonense* Rm3, *B. phenazinium* Bph12, and *Rahnella* sp. Rs11 can probably be attributed to the presence of dead or inactive cells and exopolymers, which are counted as microbial biomass by non-vital assays. Therefore, the FDA assay offers a better alternative to non-vital staining to select biofilms with high P-solubilizing ability.

A previous report showed that organic acids play an important role in biofilm formation and EPS production (Wagh et al. 2014). P-solubilizing biofilms were shown to produce organic acids, mainly gluconic acid, described as being one of the principal acids produced by planktonic hyphobacteria and mycorrhizobacteria (Taktek et al. 2015). Organic acids are responsible for releasing soluble P (Kpomblekou and Tabatabai 1994) required for bacterial growth and consequently biofilm and EPS production (Fang et al. 2008; Mendrygal and González 2000; Rinaudi et al. 2006; Wielbo and Skorupska 2008). Consistently, it has been reported that some rhizobacteria develop biofilms as physiological mechanisms for coping with P-starvation due to low concentration of free P in soils (Danhorn et al. 2004). Hence, it can be hypothesized that PSB regulated organic acids production and P-solubilization to maintain and develop biofilm. However, the reactions taking place when living phosphate-solubilizing microorganisms are using PR as sole P source are very complex, and many physicochemical and biological reactions are taking place (Richardson and Simpson 2011) including mobilization of P by efflux of protons and organic acids, immobilization of soluble orthophosphates by growing cells or its reaction with cations forming de novo poorly soluble phosphates, and the catabolism of organic anions by cells. Further work is required to elucidate how these complex reactions influence the molecular mechanisms involved in the formation of biofilms (Donlan and Costerton 2002).

SEM observations of *B. anthina* Ba8 on Quebec PR surfaces depicted the development of compact and relatively smooth structures contrary to what occurs on hydroxyapatite particles. This could suggest that hydroxyapatite surface is not suitable for biofilm development and/or lead to the formation of an exopolymeric matrix that was not enough abundant to resist fixation and drying. Likewise, no interaction between *B. anthina* Ba8 and AMF *Ri* was observed when KH_2_PO_4_, a solid phase-free control, was used as a soluble source of P. This could be due to the absence of solid particles on which bacterial cells can adhere and form biofilm or to the soluble P availability in the distal compartment. Indeed, we suggest that soluble P would be rapidly translocated to the transformed chicory roots or consumed by planktonic bacteria to ensure their own growth. Accordingly, it has been reported that high levels of soluble P can reduce AMF extraradical mycelium colonization and development (Plenchette et al. 1983), and then fungal exudates and other molecules taken up by bacterial cells as nutrients in the distal compartment could be reduced.

Our observations suggest that more viable cells in the biofilm formed on PR would have a great impact on P-solubilization in soils. This result concurs with a previous study showing that phosphate dissolution on the surface of sulfidic waste rocks mixed with natural PR was intimately linked to the presence of microbial biofilms (Ueshima et al. 2004). Cells remained attached to the rock surface through the bacterial exopolymers, which were an integral part of the biofilms. The authors suggested that biofilms would act as a physical and chemical barrier to limit the extent of pyrite oxidation and contributed to attenuate acid mine drainage (Ueshima et al. 2004).

In soil, phosphate-solubilizing bacteria that strongly adhere to PR surfaces can interact synergistically with other microorganisms such as fungi. A review of interactions between bacteria and both saprophytic and mycorrhizal fungi (Ul Haq et al. 2014) illustrates that soil bacteria can adhere to fungal cells, stimulate fungal exudates production, and also form a biofilm along the fungal hyphae. The resulting biofilms can be used for potential applications in the field (Seneviratne et al. 2008). For example, biofilm-consortia were efficiently able to mobilize poorly soluble phosphate in PR and to enhance P availability and soil fertility (Jayasinghearachchi and Seneviratne 2006).

Reports of interactions between microorganisms and mycorrhizae suggested that AMF may not associate with soil bacteria randomly but rather in a hierarchical system of mutual preferences (Andrade et al. 1998a). An experiment conducted to determine the effects of mycorrhizae on the selected groups of soil microorganisms in water-stable soil aggregates showed that a greater number of bacteria namely PSB can be associated with the aggregated soil fractions (Andrade et al. 1998b). The authors reported that the components of mycorrhizae provide favorable and protective microsites for bacteria that would enhance both plant growth and soil stability.

In the current study, we demonstrated that *B. anthina* Ba8 actively colonized the surface of *Ri* fungal hyphae in the presence of Quebec PR, while when Quebec PR was substituted by hydroxyapatite, *B. anthina* Ba8 adherence to AMF *Ri* hyphae was unsuccessful and bacterial cells were scattered. In the presence of *B. anthina* Ba8 and the hyphae of the AM fungus *Ri*, the significant mobilization of P from Quebec PR (Taktek et al. 2015) can be increased due to the capacity of Ba8 to colonize the surface of AMF *Ri* hyphae. Active attachment of PSB may be favored by Quebec PR surface and enhanced by root and AMF *Ri* hyphae exudations. As observed by Kaiser et al. (2015), AMF may act as a rapid hub for translocating fresh carbon, derived from recent plant photosynthates, to hyphae-associated soil bacterial communities which are generally composed of specific assemblages of species that differ from those in the bulk soil. This fact can be exploited to develop sustainable agriculture technologies, since phosphate-solubilizing bacteria could rapidly colonize the hyphosphere of the AMF *Ri*, efficiently mobilize P from natural PR and promote plant growth. Nevertheless, further research is needed to explain the mechanisms involved in Ba8-AMF *Ri* attachment in the presence of igneous PR. In line with these results, Ul Haq et al. (2014) reported that taxa that belong to the *Burkholderia* genus were the most abundant colonizers of fungi in soil. *Burkholderia terrae*, a migration-proficient bacterium, was able to develop a biofilm on the growing hyphae of the non-mycorrhizal fungus *Lyophyllum* sp. strain Karsten, leading to bacterial translocation and growth in novel microhabitats in soil (Warmink et al. 2011; Warmink and van Elsas 2009). The authors suggested that the migration promoting effect of *B. terrae* in conjunction with fungi plays key facilitating roles for other beneficial bacteria in soil. Furthermore, bacterial colonization of arbuscular mycorrhizal hyphae (Scheublin et al. 2010; Toljander et al. 2006) and biofilm formation on ectomycorrhizal hyphae have also been described (Nurmiaho-Lassila et al. 1997; Sarand et al. 1998). *Pseudomonas* isolates, belonging to the Proteobacteria phylum, as *Burkholderia*, showed a greater attachment to hyphae than other bacterial strains. Recently, Frey-Klett et al. (2007) demonstrated that the survival of *Pseudomonas fluorescens* is significantly enhanced by the presence of the ectomycorrhizal fungal species *Laccaria bicolor*, from which the bacterium was isolated. They observed that *P. fluorescens* adheres to the hyphae of different ectomycorrhizal fungi and developed in vitro biofilm-like structures on *L. bicolor* hyphae. The common feature between this last report and the present work is the fact that bacterial strains develop a biofilm on the hyphae of specific mycorrhizal fungi from which they were initially isolated. Our results are therefore in agreement with the previous postulate (Toljander et al. 2006), suggesting the presence of a bacteria-AMF specificity.

In this work, we demonstrated that *B. anthina* Ba8, isolated from the hyphosphere of AMF *Ri* (Taktek et al. 2015), can strongly adhere to abiotic and biotic surfaces and allow a higher solubilization of phosphate than other isolated PSB. More work will be needed to determine if the highest efficiency of hyphosphere bacteria can be generalized.

In a 2-month greenhouse trial performed in a loam soil (P Mehlich III 9.5 mg/kg^−1^; pH 6.24), inoculation of maize (*Zea mays* L. cv. Focus) with the AMF *Ri* DAOM198197 and *B. anthina* Ba8 significantly increased plant shoot fresh and dry matter yields and P uptake as compared to control not receiving Ba8, when the recommended rate of superphosphate or the low reactivity Quebec PR were used (unpublished results). Inoculation trials under field conditions merit further investigation to determine if these beneficial microorganisms can have practical applications.

## Electronic supplementary material

Below is the link to the electronic supplementary material.

Additional file 1 Conceptual drawing of the approach used to study the interaction between extraradical *Rhizoglomus irregulare* DAOM 197198 hyphae and *Burkholderia anthina* Ba8 using a two-compartment Petri dish. (DOCX 303 kb)
