# Supplementary material for: Igneous phosphate rock solubilization by biofilm-forming mycorrhizobacteria and hyphobacteria associated with Rhizoglomus irregulare DAOM 197198
Source: Mycorrhiza. 2016 Aug 19;27(1):13–22. doi: 10.1007/s00572-016-0726-z (PMC5203815; doi:10.1007/s00572-016-0726-z)
Supplement: Supplementary file 1 — Conceptual drawing of the approach used to study the interaction between extraradical Rhizoglomus irregulare DAOM 197198 hyphae and Burkholderia anthina Ba8 using a two-compartment Petri dish. (DOCX 303 kb) [file 572_2016_726_MOESM1_ESM.docx]

**Additional file 1**

**Igneous phosphate rock solubilization by biofilm-forming mycorrhizobacteria and hyphobacteria associated with *Rhizoglomus irregulare* DAOM 197198**

**Salma Taktek^1^, Marc St-Arnaud^2^, Yves Piché^3^, J.-André Fortin^3^ and Hani Antoun^1^**

^1^ Centre de recherche en innovation sur les végétaux and Département des sols et de génie agroalimentaire Faculté des Sciences de l’agriculture et de l’alimentation, Université Laval, Québec, Qc, Canada

^2^ Institut de recherche en biologie végétale, Université de Montréal and Jardin botanique de Montréal, Montréal, QC, Canada

^3^ Centre d’étude de la forêt and Département des sciences du bois et de la forêt, Faculté de foresterie, de géographie et de géomatique, Université Laval, Québec, Qc, Canada

**Corresponding author.** Hani Antoun, Centre de recherche en horticulture, Université Laval, Québec, Qc, G1V 0A6, Canada. hani.antoun.1@ulaval.ca


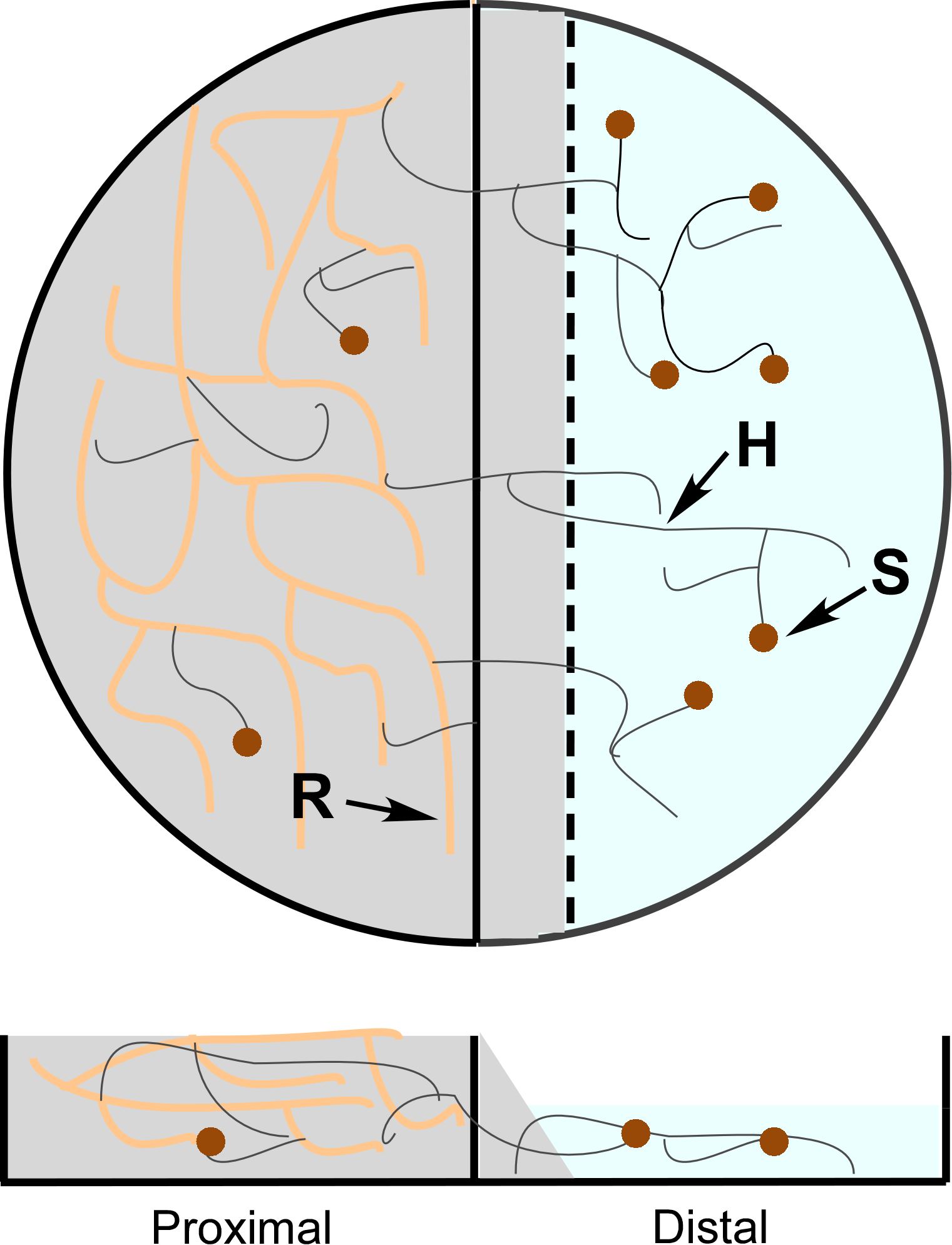


**Supplementary Figure 1.** Conceptual drawing of the approach used to study the interaction between extraradical *Rhizoglomus irregulare* DAOM 197198 (*Ri*) hyphae and *Burkholderia anthina* Ba8 using a two-compartment Petri dish. Initially *Ri* was grown at 28°C on roots of chicory (*Cichorium intybus*) transformed with the tumor-inducing plasmid T-DNA on a solid medium (grey) in the proximal compartment. After approximately 3 weeks, when the hyphae colonized the solid medium in the distal compartment a 1 cm wide hyphal zone was kept in the distal compartment and the rest was discarded and replaced with a liquid (blue) minimum medium as described in material and methods. After another 3 weeks required for hyphae to colonize the liquid distal compartment Quebec phosphate rock (ground to <150 µm) and 10 µL Ba8 bacterial suspension containing 10^6^ CFU were added in the distal compartment. After an additional 6 weeks of incubation hyphae with attached bacteria were collected and processed for SEM observations. R: roots, S: spores and H: hyphae of *Ri*.
